# Modelling the system dynamics of household food, water, and energy nexus effects

**DOI:** 10.1016/j.heliyon.2024.e25886

**Published:** 2024-02-12

**Authors:** Hanyu Liu, Wanglin Yan, Hikaru Kobayashi

**Affiliations:** a Graduate School of Media and Governance, Keio University, 5322 Endo, Fujisawa, 252-0882, Japan; b Faculty of Environment and Information Studies, Keio University, 5322 Endo, Fujisawa City, Kanagawa Prefecture, 252-0882, Japan; c Research Center for Advanced Science and Technology, The University of Tokyo, 4-6-1 Komaba, Meguro-ku, Tokyo, 153-8904, Japan

**Keywords:** Household food-energy-water nexus, Nexus effect, System dynamics, Trade-offs and synergies of interactions

## Abstract

The food-energy-water nexus (F-E-W) serves as a crucial resource for the sustainability of households, while the efficiency of resource use largely depends on our understanding and management of the nexus including all three factors. Limited research has been conducted on this topic thus far because of the increasing complexity of home technologies and data availability. This study develops an evidence-based system dynamics model for assessing the synergy and trade-offs of the household F-E-W. By applying the system dynamics (SD) methodology, the FEW consumption and generation originating from home appliances were modelled and simulated. The model was applied to an eco-house in Tokyo, and its efficacy was validated with one-year hour-based observations of a home energy management system (HEMS). The findings revealed that water-related and food-related energy use accounted for approximately 55% of the total energy use. In addition, water-related energy use showed high uncertainty, suggesting a management potential of approximately 24% for reduction, and was significantly correlated with household carbon emissions. Moreover, this result verified that the effective management of household energy consumption requires the adept manipulation of the diverse array of energy sources employed for air and water heating, while HEMSs could play a key role in implementation.

## Highlights

• Proposed a household FEW nexus model incorporating resource self-production systems.
• Data-based modeling using data collected from home energy management system.
• Captured the dynamics of interactions between FEW consumption at the household level.
• Assessed the impact of behavior and appliances on household FEW nexus by scenario and sensitivity analysis.
• Explored the nexus effect on household FEW management and decarbonization.

## 1 Introduction

The urban household sector accounts for significant quantities of food, water, and energy (FEW) consumption, making it an important component in resource management [1,2]. For instance, the household sector in Tokyo consumes 31.7% of the city's total energy use and 89% of its total water use. In addition, FEW demand are projected to increase by 55%, 80%, and 60%, respectively, by 2050, posing challenges for maintaining a sustainable supply [3,4].

Household electricity consumption is considerably attributed to water and food use [5]. The interdependencies among food, energy, and water in households have been comprehensively examined in the integrated framework of the food-energy-water so-called nexus approach for reducing inefficient resource use due to interdependencies between sectors [[6], [7], [8]].

The methodology of the nexus approach is evolving with its increasing popularity in the field [9]. Researchers have proposed frameworks to assess the resilience and sustainability of cities under the assumption of an F-E-W nature [[10], [11], [12]]. Many studies have investigated the interactions among F-E-W in industrial production [[13], [14], [15], [16]], especially resource flows through sectors in F-E-W supply chains. The relationship between the external and internal consumption of F-E-W in households has also been examined. For instance, the flow of FEW resources among the sectors triggered by households has been assessed by using substance flow analysis and input-output analysis [17]. The vulnerability of households during disasters, affected by disruptions to the FEW infrastructure, has been analysed with structural equation modelling [12]. Furthermore, given the intricate and interconnected nature of the nexus, system thinking is widely accepted for analysing and modelling interactions [18]. For instance, the ▣interdependences of agricultural, energy and hydrological systems at a large scale were captured by an integrated system dynamics model [13,15].

While many studies have focused on the environmental load of individuals and families by accounting for the footprint of urban food, water and energy consumption [11,19], investigations of the interactions of F-E-W at the household scale are very limited [20]. This limitation can be attributed to the lack of detailed data on the resource use of end-use appliances, which reflect the consumers’ habits and resource use efficiency of different appliances [2,21].

Uniquely, a dynamics-based model was developed to address the interactions among food, water and energy at the end-use level at a household scale with data collected from 419 households in Duhok [22]. This study also provided a fundamental framework of the F-E-W and a data reference for subsequent studies at a household scale. However, this survey lacked an appropriate temporal resolution, which hindered the assessment of dynamic changes in the interactions and nexus effect of F-E-W.

Nexus effects are defined as the interconnected impacts resulting from the interactions among the food, energy and water systems, encompassing consequences such as resource availability, efficiency, sustainability, resilience, and environmental impacts [23]. A nexus approach can provide significant insights for effective and sustainable resource management by enhancing synergistic effects while avoiding trade-offs between F-E-W.

Nexus effects are the culmination of dynamic interactions affected by multiple factors. These factors have been classified as household demographics, seasonal variation, consumer behavior, and appliance efficiency in a previous system dynamics model [24]▣. However, the house features (i.e., floor area, insulation performance) were not incorporated into the household F-E-W model. The factors related to house structure, such as material thermal properties, were considered when investigating changes in the water and energy practices of households [25]. Furthermore, previous models have generally failed to consider the resources of household self-production, such as popular photovoltaic panels. Sui et al. [26] assessed the performance of an eco-house with grid-connected photovoltaics influenced by weather-related factors (i.e., temperature, radiation) only in terms of its electric and heat loads.

Based on the factors considered, the average dynamic consumption of FEW per household was predicted by the household F-E-W dynamics model. In addition, scenarios were designed to simulate the effect of the regulation measures on the reduction of F-E-W and carbon emissions [27]. Since many previous studies have focused on long-term dynamics interactions at the household level, research assessing the dynamics nexus effect of F-E-W inside a house is still lacking.

To fill this gap, this study developed an evidence-based system dynamics model to investigate the dynamics of FEW demands, interactions, and CO_2_ emissions originating from end-use appliances. The household resource self-production system was internally incorporated into model, while the factors related to weather and house features were treated externally. The model was validated by one-year hour-based observations of the home energy management system the FEW nexus of an eco-house in Tokyo. As a result, the synergies and trade-offs of the F-E-W nexus effects were quantitatively assessed, and the performance of the entire home system was discussed. The developed household FEW nexus model can support the formation of integrated FEW management strategies for each individual and serve as a basis for analysing the policy effect for the sustainable management of F-E-W supplies and demands in urban household sectors.

## 2 Developing the household FEW nexus model

**Fig. 1:**
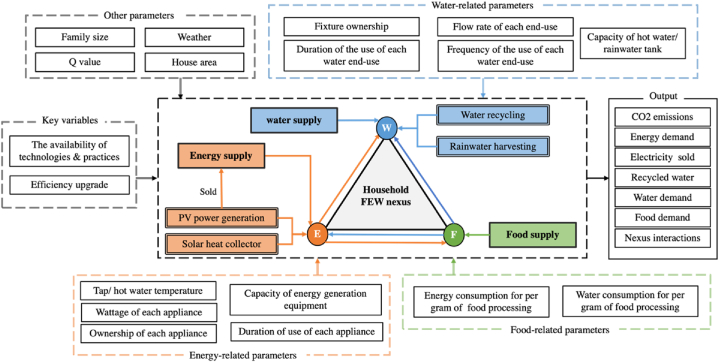
The structure of the household FEW nexus model.

**Fig. 2:**
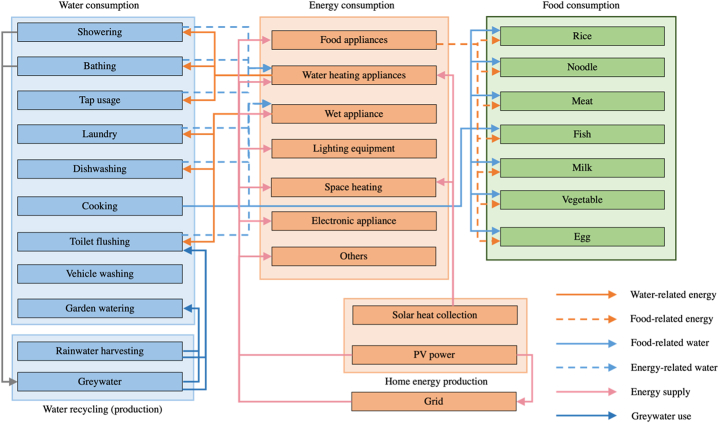
Modelling the interactions between household FEW consumption and supply.

The structure of the household food-energy-water nexus models is illustrated in Fig. 1, highlighting the interactions of various components. A bottom-up approach was employed to investigate the interactions of the nexus by disaggregating the FEW uses into specific end uses. Based on previous studies, this model incorporated the adoption of household resource self-production systems, including photovoltaic systems, solar thermal systems, water recycling systems, and rainwater harvesting systems. Moreover, the model facilitated the assessment of the contribution of each end use to total consumption, identifying the end uses with the highest levels of resource consumption and interactions [28]. Consequently, this model can provide support for integrating household resource management while simulating the dynamic interactions of the FEW nexus at the household level. An important aspect of this model is its ability to not only quantify the consumption of FEW but also capture the intricate interactions triggered by each FEW end use. The intricate interactions between the different components of the FEW system are visualized and presented in Fig. 2.

Since resource self-producing systems have been incorporated into the model, weather-related parameters such as radiation and temperature were also included to enable a comprehensive analysis of their impact on the performance of the resource self-production systems. Furthermore, building-related factors, including floor area and Q-value, were incorporated to provide a more accurate assessment of the heating load within a house.

The model structure presented here is designed to be applicable to general households, with the flexibility to adjust the values of the relevant parameters based on the specific conditions of different households. For instance, the average household FEW nexus conditions can be assessed by determining input variables and parameters based on the average consumption patterns of each FEW end use within the urban household sector of the city. In this study, the model was applied to an eco-house equipped with a resource self-production system and home energy management system (HEMS). To validate the simulation results of the model, data collected from the HEMS were utilized. The inclusion of HEMS data allows us to assess the reliability and accuracy of the model's performance in a real-world setting.

System dynamics modeling is an efficient methodology to address dynamically complex problems by adopting a system approach and social learning approach [29]. It posits that systems evolve through various states influenced by the interplay of stocks and flows [30]. Consider the example of the state variable is the demand for grid electricity in the study of household energy demand. The variable changes over time and is influenced by inflows (such as the power supplied to the appliance) and outflows (including Self-produced electricity use). The approach allows us to model and simulate the dynamic behavior of electricity consumption, considering factors like appliance usage, and energy-efficient technologies. The equation of the state variable is expressed as:

(1) $$E_{t+1}=\int_{t}^{t+1}\left[I_{s}-R_{s}\right]ds+E_{t}$$

Where $E_{t+1}$, $E_{t}$, $I_{s}$, $O_{s}$ denote the demand for grid electricity at time t+1, the demand for grid electricity at time t, the increase rate of grid electricity demand at time s, the reduction rate of grid electricity demand at time s, ▣, respectively.

Vensim® DSS was used to construct the system dynamics model and perform the simulations. ▣More detailed explanations can be found in the Appendix.

### 2.1 Household energy subsystem modelling

The energy subsystem was partitioned into two key components: the energy demand system and the energy self-production system. Within the energy demand system, the household's energy demand was categorized according to distinct end uses, encompassing heating, water heating, lighting, food processing, electronics, wet appliances, and other miscellaneous purposes. Table 1 provides a breakdown of the various appliances associated with each energy end use. Conversely, the energy self-production system within the household model encompassed two pivotal elements: a photovoltaic system (PV) and a solar thermal system, each contributing to the overall production of energy within the household context.

**Table 1:** Summary of energy end uses and the related appliances.

Appliances	Classification
PV, solar heat collector system	Energy generation equipment
Lights	Lighting appliances
Dishwasher, warm water toilet, washing machine	Wet appliance
Others	Other appliances
Computer, TV, etc.	Electronic appliances
Air conditioning, solar heat collector system	Heating appliances
Refrigerator, oven, Microwave oven, gas oven,	Food appliances
Gas water heater, solar heat collector system	Water heating appliances

The interrelationships among energy appliances are visually represented in Fig. 2. Typically, the electricity generated by the photovoltaic (PV) system is allocated to power different household electrical appliances. Any surplus electricity produced can be exported to the grid, while the grid supplements any shortage to meet the electricity demand. Conversely, the solar thermal system primarily utilizes the collected thermal energy for space heating during winter or for water heating throughout the year, with a priority given to meeting space heating demands. Furthermore, the intricate interactions between FEW end uses and their respective supply at the household level are illustrated. The arrows indicate the FEW uses associated with each end-use appliance and highlight the interactions within the model. Notably, the figure showcases the water and energy uses prompted by wet appliances and water heating, as well as the electricity, gas, and water uses triggered by food-related activities.

To calculate the energy demand of each appliance specified in Table 1, the model adopted the equations proposed by Hussien et al. [22]. These equations encompass factors such as appliance wattage, duration of usage, and ownership of each appliance. To improve the model's ability to capture the energy demand patterns exhibited by energy appliances, the model's accuracy was improved by considering the average resource demand for each end-use appliance per month.

Additionally, activities involving showering, bathing, and faucet usage contribute to hot water consumption, thereby contributing to the energy demand for water heating. The energy required for water heating is influenced by various factors, including the temperature of inflow and outflow water and the specific appliance employed [31,32]. Moreover, seasonal variations in household water consumption and the temperature of inflow water impact the energy demand for water heating [33].

Furthermore, weather conditions and seasonal variation exert significant influences on the performance of household energy self-production systems. Consequently, the energy consumed for water heating in households equipped with distributed energy systems may exhibit greater complexity and variability. The detailed system dynamics model for each component can be found in the Appendix.

### 2.2 Household water subsystem modelling

Within the water subsystem, household water use is classified into two components: the water demand system and the water recycling system. The household water consumption system is further segmented into various end uses, encompassing showering, tap usage, laundry, dishwashing, food processing, and toilet flushing. Moreover, the household water recycling subsystem consists of a rainwater harvesting system and a greywater recycling system. In terms of the water recycling system, two separate storage tanks are implemented for collecting rainwater and recycling shower water. Subsequently, the collected water is subjected to purification processes for subsequent utilization in non-food applications, such as toilet flushing. The recycled shower water is referred to as greywater within the model. The quantities of rainwater and greywater collected are contingent on the capacity of the water storage tank, whereas the utilization of greywater depends on the demand for toilet flushing. The calculation of water use for each water appliance incorporates key parameters such as the flow rate specific to each end use, water fixture ownership, and frequency of water use, as shown in Fig. 1.

### 2.3 Household food consumption subsystem modelling

The food consumption subsystem encompasses a range of food categories, namely, cereals, meat, seafood, eggs and milk, vegetables, fruits, fats, and oils. The per capita consumption quantities for each food category were derived from Japanese household consumption statistics, as presented in Table 2.

**Table 2:** Summary of food categories and commodities.

Commodities	Food categories
Rice, bread, noodles, other cereals	Cereals
Beef, chicken, pork, lamb, synthetic meat, ham, sausage, bacon	Meat
Fish, shellfish, dry fish	Seafood
Eggs, milk, butter, cheese	Eggs and milks
Cabbage, onion, cucumber, carrot, Chinese cabbage, etc.	Vegetables
Apple, banana, pear, strawberry, etc.	Fruits
Edible oil, tea, condiments, etc.	Fats & oil

To accurately estimate the water use associated with food processing, the model incorporated pertinent parameters, such as the quantity of water and energy used per unit of food. These parameters are essential for determining water and energy use during food processing. The data concerning food-related energy and water use were sourced from Januar [34].

### 2.4 Impacts of weather and seasonal variation on energy self-production

To comprehensively analyse the influence of weather-related factors on the performance of household energy self-production, we investigated three key weather-related variables that impact the performance of the photovoltaic system and solar thermal systems. First, we considered the daily solar radiation, which denotes the total solar radiation energy received by a horizontal surface per unit area. Second, the daily average temperature was considered, which represents the mean temperature calculated from 24 hourly observations spanning the time period from 1:00 to 24:00. Finally, we incorporated the four distinct seasons, namely, spring, summer, autumn, and winter, as dummy variables. To assess the effects of these weather-related factors, we estimated the following equations with the daily electricity generated by the PV system and the daily heat generated by the solar thermal system as the dependent variables.

These equations were formulated to capture the relationship between weather-related variables and energy generation:

(2) $${EPV}_{i}=\alpha_{0}+\alpha_{1}{RAD}_{i}+\alpha_{2}{ATEM}_{i}+\alpha_{3}S_{1}+\alpha_{4}S_{2}+\alpha_{5}S_{3}+\varepsilon_{i}$$

(3) $${HSHS}_{i}=\beta_{0}+\beta_{1}{RAD}_{i}+\beta_{2}{ATEM}_{i}+\beta_{3}S_{1}+\beta_{4}S_{2}+\beta_{5}S_{3}+\delta_{i}$$

where ${EPV}_{i}$ and ${HSHS}_{i}$ are the daily electricity generated by the PV system and the daily heat generated by the solar heat collector system on day i, respectively; ${RAD}_{i}$ and ${ATEM}_{i}$ are the daily solar radiation and the average temperature on day i, respectively; $S_{1}$, $S_{2}$, and $S_{3}$ are spring, summer, and autumn, respectively, selected as dummy variables; and $\varepsilon_{i}$ and $\delta_{i}$ are error terms.

Based on the equations, the impact of weather-related factors on household energy production can be better understood, contributing to a more comprehensive understanding of the dynamics and performance of energy self-production systems under different weather conditions.

## 3 Model application

The developed model described in this study exhibits the potential for application across various scales, encompassing both individual households and regional household sectors. By employing this model, it becomes feasible to scrutinize FEW use for each specific household end use. Moreover, the model enables the evaluation of the effects stemming from the adoption of resource self-production systems (e.g., photovoltaic systems, solar thermal systems, water recycling). This study endeavours to track the interactions among FEW use induced by individual end uses, unveiling their dynamics and thus paving the way for proposing strategies for integrated FEW management at the household level.

### 3.1 Case study

The developed model was applied using data collected from an eco-house situated in Tokyo, Japan. Over the past five years, Tokyo has had an average temperature of approximately 16.5 °C, with an annual sunshine duration of approximately 2000 h and an average daily sunshine intensity of approximately 13.4 MJ/m2. The region also receives an annual precipitation of 1715.5 mm. The eco-house incorporates various energy self-production systems, including a photovoltaic (PV) system for electricity generation and a solar thermal system for space heating and water heating. Energy consumption in the eco-house has been improved through the use of more efficient appliances such as refrigerators, air conditioners, and lighting. In addition, the eco-house implements a water recycling system in conjunction with a rainwater harvesting system. Rainwater and collected bath water undergo purification processes and are utilized for toilet flushing purposes. Furthermore, the eco-house has an area of approximately 160 m^3^, boasts a Q-value of 2.7, and accommodates a total of 4 family members. The eco-house information is summarized in Table 3.

**Table 3:** The basic information on the eco-house.

Item	Information	
Location	Tokyo	
Climate	Radiation	13.4 MJ/m^2^/Day
	Temperature	16.5 °C/Day
	Precipitation	1715.5 mm/Year
Area	160 m^2^	
Family size	4	
Heat loss coefficient	2.7 W/m^2^K	
Appliance	Air conditioner, dishwasher, lights, microwave, oven, refrigerator, warm water toilet, washing machine, others	
Resource self-production system	Photovoltaic system, solar thermal system, water recycling system, rainwater harvesting system	

### 3.2 Data collected in the eco-house

**Fig. 3:**
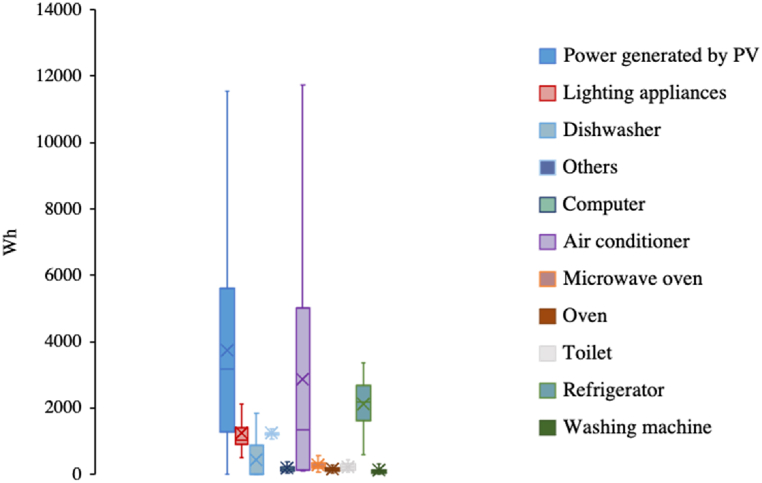
Electricity consumption of appliances and PV power generation.

In relation to energy, a home energy management system was implemented in the eco-house, consisting of 17 sensors to assess the electricity consumption of each appliance as well as the amount of electricity generated by the photovoltaic system. Fig. 3 shows the daily electricity consumption of high-power appliances and electricity generation throughout 2019. The air conditioner had the highest electricity consumption, followed by the refrigerator and lighting. Notably, sporadic periods of heightened appliance usage and PV generation exerted an upwards influence on overall electricity consumption and generation levels. In addition, the gas consumption in 2019 totaled 208 m^3^, with notably reduced usage during the summer compared to other seasons. Regarding water, tap water consumption in 2019 amounted to 182 m^3^, exhibiting consistency across seasons. Given the unavailability of specific food consumption data for eco-houses, the per capita consumption of various food categories was derived from Japanese household expenditure statistics. Further details on the data can be found in the Appendix.

## 4 Results

### 4.1 Validation of the household FEW nexus models applied to the eco-house

The results of the household FEW nexus model were compared with available historical data. Historical data for water and energy were collected from the HEMS, and historical data for food were obtained from the statistics of Japanese household consumption. The comparison between the results of the model and the available historical data of FEW consumption is shown in Table 4. The results showed that the simulation results of the model were close to the historical data. However, the simulated value of heat for water heating was slightly lower than the historical value. This difference may be due to differences in the performance of the OM system for heat collection and heat use allocation triggered by weather variables, including temperature and radiation, which lead to differences between the simulated and historical values of related variables, such as gas for water heating and heat for heating. In addition, since the household food consumption simulation was based on national average household consumption data, differences from actual household food consumption result in differences in food-related energy and water use variables, such as external gas supply and household water demand.

**Table 4:** Comparison of model results with historically measured results.

Description	Unit	Model result	Historical data	Reference
Electric load	kWh/y	4364	4594	
Grid power supply	kWh/y	3323	3624	Eco-friendly houses are connected horizontally [35]
PV power generation	kWh/y	1383	1355	
PV power sold	kWh/y	394	385	
External gas supply	kWh/y	2663	2662	
Gas for water heating	kWh/y	1648	1603	
Heat for water heating	kWh/y	1711	1835	
Water supply	m [3]/y	183	182	

### 4.2 Sensitivity analysis of household nexus interactions

To indicate how the usage of end uses influences household FEW demands, a sensitivity analysis was conducted. This analysis considered the variation range of input parameters related to consumer behaviours (i.e., the duration of each electric appliance) that affect each FEW end use as its standard deviation below and above its average value. The change in the minimum and maximum FEW values of each input parameter was individually quantified while maintaining all other input parameters at their baseline value [36].

**Fig. 4:**
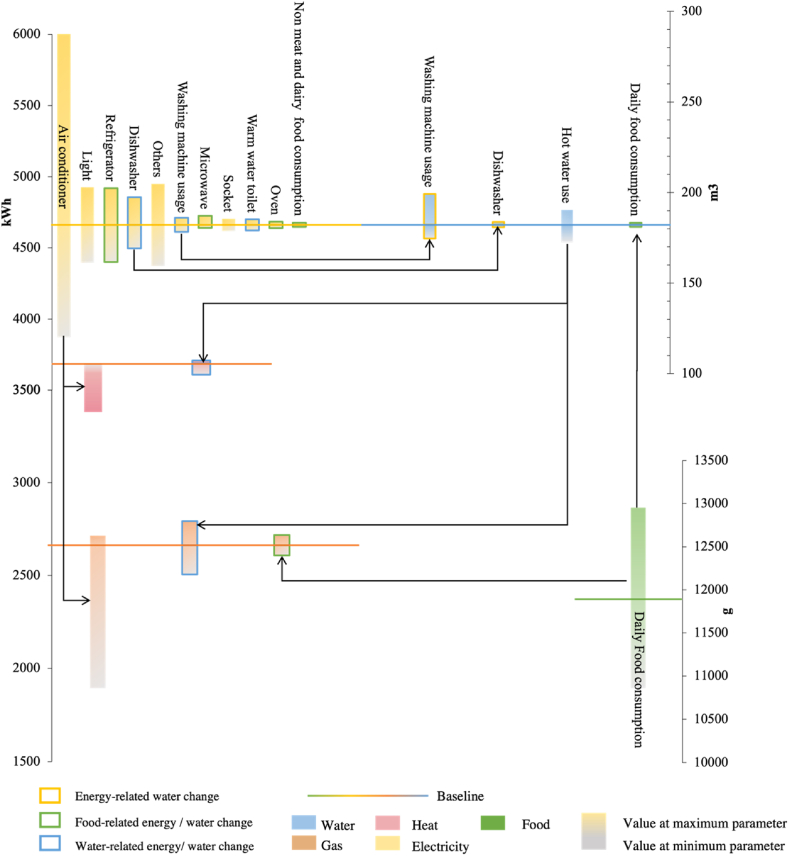
Sensitivity analysis of household energy and water use estimation for each FEW end-use consumption.

Fig. 4 shows the sensitivity of household energy and water use to each FEW end use. In terms of household energy, the highest sensitivity was from the use of air conditioning, with the sensitivity to electricity consumption estimation ranging from approximately −17% of the lowest to 33% of the highest estimated consumption for the base case. Due to the interactions between resources, the duration of air conditioner use had a significant impact on heat and gas use. On the one hand, an increase in heat used for heating and a decrease in heat used for water heating can be attributed to the reduction in air conditioner use, thus increasing the gas consumption used for water heating. On the other hand, as more electricity is used for heating by the air conditioner, there is more heat available for water heating and a lower consumption of gas for water heating. Second, the duration of the usage of lighting and refrigerators all contributed to the sensitivity of the electricity consumption estimations, accounting for approximately ±7% of the base-case estimated consumption. Additionally, the change in hot water demand triggered by bath water use contributed to the sensitivity of the gas demand estimation, accounting for ±6% of the base-case estimated consumption. Other FEW end-use changes had lower impacts on the energy consumption estimates (under ±3% of the base-case estimated demand).

In terms of water use, washing machine use had the largest impact on water use estimation, with the sensitivity to water use estimation ranging from approximately −4% of the lowest to 10% of the highest estimated. Second, hot water demand contributed to the sensitivity of water use estimation, accounting for approximately ±6% of the base-case estimated use.

Additionally, there was a strong correlation between the nexus interaction and the sensitivity of household energy and water consumption. The cumulative sensitivity to estimated energy consumption ranged from approximately −45%–30% of the base-case estimated consumption. In contrast, cumulative water-related and food-related energy consumption resulted in a sensitivity to estimated energy consumption ranging from −22% to 15% of the base-case estimated consumption. Moreover, the sensitivity of energy-related and food-related water use to water use estimates accounted for approximately ±18% of the base-case estimated use. -

### 4.3 Uncertainty of household FEW nexus

The Monte Carlo technique was used to assess how the output of the household FEW nexus model affected the uncertainty of the input parameters [37,38]. Random values were selected from the distribution of possible values of the input parameters to calculate the output of the model, and the process was iterated a certain number of times to obtain the probability distribution of the model output. With the HEMS data, the study assessed the dynamics of the FEW nexus due to the uncertainty in the usage of each FEW end use.

**Fig. 5:**
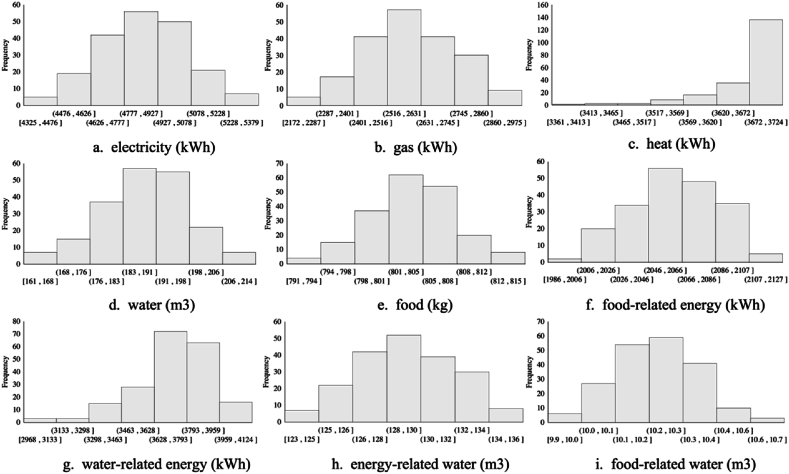
Probability distributions of the household FEW nexus.

The probability distributions of the household FEW nexus are shown in Fig. 5. The analysis showed that the uncertainty in the electricity demand estimates ranged from 4018 kWh to 4776 kWh and was of higher probability between 4397 kWh and 4586 kWh. The uncertainty in the gas demand estimates was higher than the estimated electricity demand, ranging from 2245 kWh to 3117 kWh. The estimated heat use was more concentrated, mostly between 3135 kWh and 3196 kWh. However, there was more uncertainty in the estimated water-related heat use and heat used for heating. In terms of water, the uncertainty of the estimated water demand ranged from 167 m3 to 212 m3 and was of higher probability between 178 m^3^ and 195 m^3^. In addition, the uncertainty in the estimated CO2 emissions triggered by FEW use ranged from 2180 kg to 2557 kg, which was significantly less than the average carbon emission level of Japanese households. In terms of interactions between FEW uses, including food-related energy, water-related energy use, and energy-related water use, significant effects on FEW use were shown.

### 4.4 Scenario analysis

The eco-house has undergone renovation, progressive installation of the resource self-production system, and appliance upgrades. In this study, a scenario analysis was designed to investigate the impact of these initiatives on the household FEW nexus. The impacts of changes in food consumption and the use of water end-use appliances were included in the scenario analysis as well. The scenarios are explained in Table 5.

**Table 5:** Summary of household FEW nexus scenarios.

Scenarios	Description	Related variable
Baseline	Self-production upgrade, appliance upgrade	/
S1	No resource self-production system	The availability of each resource self-production system
S2	No electric appliance upgrade	Change rate of wattage of each electric appliance
S3	Food consumptions level in 2000, water use level in 2002 by each end-use	Change rate of water use by each end-use Change rate of food consumption Change rate of energy used for food processing
S4	Food consumptions level in 2010, water use level in 2012 by each end-use	

Some previous studies have designed appliance and behaviour scenarios to assess the range of appliance impacts on household FEW use [22,27]. In this eco-house case study, interactions in the five scenarios were analysed. In the first scenario (S1), no household resource self-production system was installed. In the second scenario (S2), no appliance updates were performed. In the third and fourth scenarios (S3 and S4), end-use appliances had different levels of food and water use. Finally, the status quo was used as a baseline. The data used in the scenario analysis can be found in the Appendix.

**Fig. 6:**
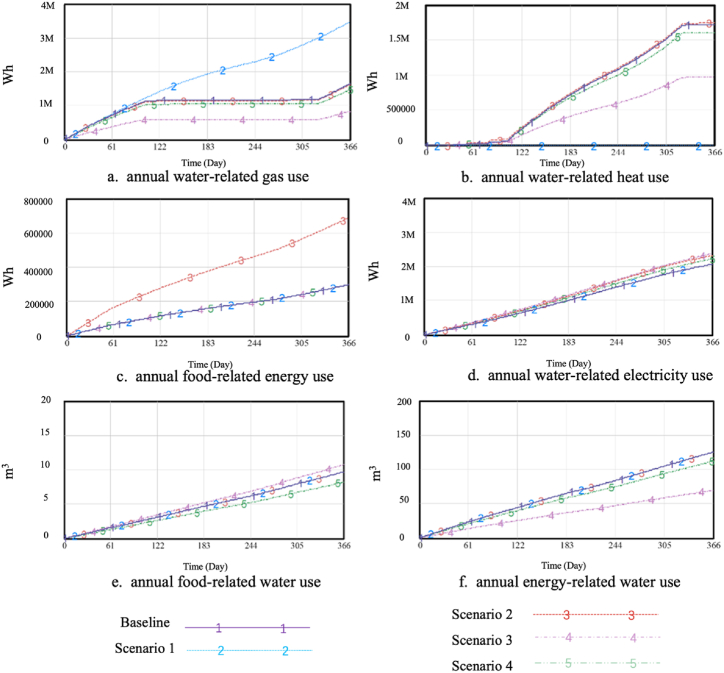
Nexus interactions in the different scenarios.

The interactions between FEW uses are shown in Fig. 6. First, appliance upgrades and the adoption of solar thermal systems made the interactions between energy and water in the home extensive. On the one hand, upgrading the appliances reduces the water-related and food-related electricity uses. In particular, the increased energy efficiency of wet appliances reduced the water-related electricity use. On the other hand, the adoption of solar thermal systems has reduced the dependence of households on the external energy supply, as evidenced by the significant decrease in water-related gas use and the heat generated by solar thermal systems as an alternative for water heating. Additionally, changes in food and water uses in scenarios 3 and 4 had a significant impact on energy use, particularly as the reduction in hot water use led to a reduction in water-related gas and heat use.

Furthermore, the integration of the PV system had a noteworthy effect on reducing the dependency on grid electricity supply. However, due to the absence of a comprehensive breakdown detailing the utilization of electricity generated by the PV system, a thorough examination of its impact on the FEW nexus cannot be explained in this study.

## 5 Discussion

### 5.1 Trade-offs and synergies in household nexus interactions: implications for resource management

The prevalence of interactions between daily FEW uses offers a perspective for integrated resource management. Previous studies have categorized nexus interactions according to resource flows, such as water-related energy or energy-related water. However, these studies have neglected to capture the essential nature of trade-offs (where one service decreases while another increases) and synergies (where both services increase or decrease together) between interactions. Trade-offs and synergies play a crucial role in the pursuit of effective resource management guided by the nexus approach [39,40]. In the context of the eco-house equipped with resource self-production system, the relationships of synergies and trade-offs between interactions become more prominent and complex due to the allocation of the use of limited productive resource.

Expanding upon the findings depicted in Fig. 4, the study examined nexus interactions from the standpoint of trade-offs and synergies. First, synergistic interactions exist between different types of resources. The use of one resource is reliant on the use of another. For instance, water heating necessitates energy use, while food processing involves both energy and water uses. Second, there are trade-offs between interactions with the same purpose of use. An example of this can be observed in an eco-house equipped with two energy sources (gas and heat) for water heating, where an increase in heat usage leads to a corresponding decrease in gas use.

Third, when a resource is limited, trade-offs arise when allocating it across different purposes. In the case of an eco-house generating electricity and heat via a solar system, the total daily capacity of self-generated energy is finite. Consequently, increasing its utilization for one purpose inevitably curtails the availability of self-generated energy for other applications. Finally, it can be further observed that there is a synergistic relationship based on the previous scenario. When the use of a particular resource is increased, the demand for other resources may decrease, thus making more resources available for other purposes. For instance, within an eco-house, heat can be used for both space heating and water heating, whereas gas can be employed only for water heating, thus establishing a synergistic relationship between gas usage for water heating and heat usage for space heating.

Once trade-offs and synergies between interactions are identified, they can be leveraged to manage household resource utilization. When the goal is to reduce emissions without altering consumption patterns and appliances, optimizing resource efficiency and assessing carbon emissions for interactions with trade-offs becomes crucial. This includes considering the carbon emissions associated with alternative energy sources and specific appliances used for heating and water heating. By considering these factors, more sustainable and emissions-conscious resource management strategies can be enabled.

### 5.2 Nexus effects on household FEW management

**Fig. 7:**
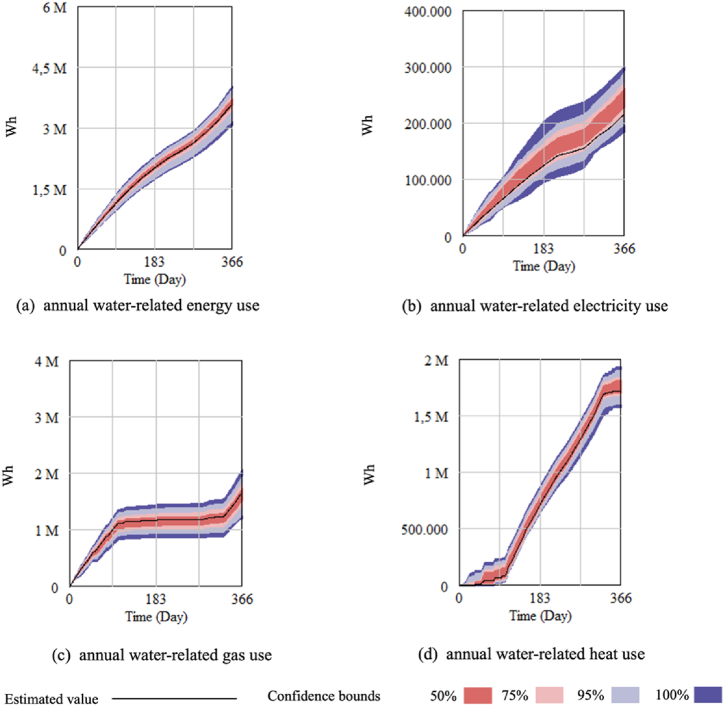
The uncertainty of water-related energy use.

Nexus effects are assessed in terms of FEW saved and environmental impacts to validate the importance of considering interactions between FEW components [27]. Water-related energy use was assessed to be the most important interaction in the house. Fig. 7 shows the nexus effect triggered by water-related energy use and its distribution at different confidence bounds. Confidence bounds for the simulation results of each output value were computed and displayed by ordering and sampling all the simulation runs. The findings showed that approximately 3500 kWh of energy use in a year was water-related in the household, including water-related heat use, water-related gas use and water-related electricity use. The higher share of the first two was because of the demand for hot water.

Managing the interactions between water and energy can be accomplished through considerations of consumer behaviour, appliance type and efficiency. On the appliance side, the utilization of household resource production appliances introduces new dynamics to these interactions. Scenario analysis revealed that adopting a solar thermal system led to extensive interactions between water and energy, reducing the dependence on the external energy supply by approximately 3150 kWh.

In terms of consumer behaviour, the study identified the potential upside and downside resulting from the uncertainty of water-related energy use driven by household behaviour. Fig. 7 illustrates the 50% uncertainty bounds, which indicate that the water-related electricity use is most likely to range between approximately 260.21 kWh (maximum) and 227.69 kWh (minimum), while the estimated water-related electricity is 216.48 kWh. This suggests a potential for increased water-related electricity use. Additionally, a trade-off was observed between water-related heat and water-related gas, with an opportunity to reduce water-related gas use by increasing the utilization of water-related heat. To support this, the capacity of the hot water tank must be expanded to accommodate greater heat use for water heating.

Moreover, during the season requiring space heating, adjustments should be made to the air conditioner in alignment with the performance of the solar thermal system to prevent excessive energy use for space heating. These measures collectively contribute to the effective management of water-energy interactions in a household context.

### 5.3 Managing the FEW nexus for decarbonization

**Fig. 8:**
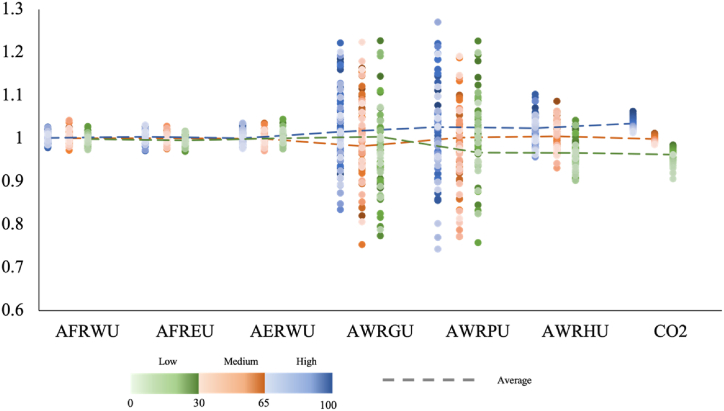
The FEW nexus at different CO2 emission levels. AERWU, annual energy-related water use; AFREU, annual food-related energy use; AFRWU, annual food-related water use; AWRPU, annual water-related power use; AWRGU, annual water-related gas use; AWRHU, annual water-related heat use.

To mitigate carbon emissions, the analysis of the FEW nexus at various carbon emission levels is essential. Fig. 8 illustrates the breakdown of CO2 uncertainty data obtained from sensitivity analysis into low, medium, and high levels. The average CO2 emissions and interactions between FEW components were calculated within each interval. The low, medium, and high intervals were determined based on the 30% and 65% quartiles of the CO2 data.

The findings indicated a strong correlation between household CO2 levels and water-related energy consumption, encompassing water-related heat, gas, and power usage. This relationship may be attributed to the relatively stable patterns of household food and water consumption. Conversely, household water-related energy consumption displayed more variability due to factors such as consumer behaviour, appliances, and weather conditions. Within the context of the FEW nexus in the eco-house, carbon emission reduction necessitates greater attention to the selection of energy sources for water heating. Enhancing the utilization of heat collected by the solar thermal system for space heating can effectively reduce electricity consumption from air conditioners, thereby improving decarbonization efforts.

However, in this scenario, reducing heat for water heating necessitated an increase in gas consumption. Moreover, the findings indicate that lowering water-related electricity use is advisable at low CO2 emission levels. Within households, diminishing water-related electricity consumption primarily entails reducing the use of wet appliances such as washing machines and dishwashers, which typically correspond to an augmented household water demand. To this end, the resource efficiency of wet appliances can be improved, simultaneously reducing resource consumption and carbon emissions.

Additionally, dietary change in affluent areas has substantial potential to reduce GHG emissions and related resource consumption, contributing significantly to environmental goals [41]. As depicted in Fig. 6 of the scenario analysis, alterations in food consumption exert a discernible influence on food-related energy and water uses. This stems from the substantial water and energy demand entailed in the preparation of certain food items, particularly meats.

### 5.4 Economic and environmental benefits

The adoption of household resource self-production system equipment like solar panels and water recycling faces a significant initial cost barrier. Subsidy programs, particularly in Japan, play a crucial role in offsetting this financial hurdle. These programs offer grants, low-interest loans, favorable housing finance terms, and tax deductions, encouraging investments in environmental technologies.

Table 6 delineates the costs and benefits of investment associated with the key system equipment employed in the case study of the eco-house. The merits stemming from such environmental investments, particularly in terms of energy generation and resource conservation demonstrate a tangible return on this initial capital investment. For example, the eco-house's efficiencies in utility cost reduction, achieved through the solar energy system and water recycling systems, can yield annual returns ranging from 195 to 260 thousand yen. Primarily, the benefits of environmental investments are rooted in the realms of energy generation and conservation. Rainwater and water recycling systems may not always represent the optimal choice for household environmental investments from an economic perspective.

**Table 6:** Cost and benefit (Ten thousand yen) breakdown of major equipment.

Item	Amount
Initial cost	488
Solar photovoltaic system	270
Solar heat collector system	138
Rainwater harvesting and water recycling system	80
Annual loan cost	1.7
Subsidy	138
Solar photovoltaic system	72
Solar heat collector system	66
Annual returns from resource savings and production	19.5–26
Annual interest relief for environmental responses	4.7
Annual Income tax relief	4.1

However, in an environmental context, these systems assume a crucial role in alleviating strain on regional water supply networks and sewage treatment facilities. By 2021, the penetration rate of piped water facilities in Japan had reached 98% [42]. Conversely, a declining population translates into reduced demands on water supply. Simultaneously, the aging state of tap water infrastructure necessitates substantial expenditures for renewal. Regrettably, these expenses can no longer be entirely covered by water utility revenue. Consequently, the implementation of household or small-scale rainwater and recycling water systems emerges as a viable strategy for mitigating the conundrum of underfunding in the water sector. Notably, water recycling systems integrated into eco-houses have demonstrated the potential to curtail water consumption by up to 42 m^3^ annually.

In Japan's recent 2050 Carbon Neutral Manifesto, a 46% reduction in CO_2_ emissions by 2030 is targeted, necessitating each household sector to attain an objective of approximately 1250 kg/year of CO_2_ emissions [43]. In 2019, the electric utility chosen by the eco-house exhibited a carbon emission factor of 0.234 kg-CO_2_/kWh, resulting in CO_2_ emissions amounting to 764 kg/year.

Moreover, considering the average household water consumption of 1 m^3^ leads to the emission of approximately 0.67 kg of CO_2_ [35], it is imperative not to underestimate the CO2 emissions associated with water usage. The incorporation of a rainwater harvesting and water recycling system within the Eco-house manifests a notable reduction in carbon dioxide emissions by 28.14 kg CO_2_. In the event of a widespread adoption of water recycling technologies by households, there exists substantial potential to significantly bolster the efforts of the water treatment sector in achieving the 2050 CO_2_ emissions reduction target.

## 6 Conclusions and policy implications

In this study, a household FEW nexus model that incorporates a resource self-production system was developed. The model serves as a tool for simulating and exploring the dynamics of interactions between FEW uses. By applying the household FEW nexus model to an eco-house, the study evaluated the impact of each end-use appliance on the total FEW demand. The uncertainty of FEW uses and their interactions were simulated. Based on these analyses, the trade-offs and synergies inherent in nexus interactions were identified, and the potential for managing household FEW uses through the nexus effect was discussed.

According to the simulation results, we found that due to the adoption of the self-production system, the interactions between household FEW uses became more extensive, especially for water-related and food-related energy uses, which accounted for approximately 55% of the total energy use. Water-related energy use exhibited high uncertainty, indicating a management potential of approximately 24% for reduction. In addition, the findings showed that water-related energy use and household carbon emission levels were significantly correlated. In the context of solar thermal system use, prioritizing the thermal demand for space heating and appropriately reducing the thermal use of water heating would contribute to reducing CO_2_ emissions.

Therefore, promoting high-energy conversion efficiency solar systems in households is crucial. Solar collectors efficiently harness sunlight to generate thermal energy, specifically for air and water heating. Additionally, significant reductions in water and energy consumption are attributed to improvements in the efficiency of electrical appliances, where incentives and regulations for upgrading household appliances can be implemented. Furthermore, the proliferation of HEMS warrants attention, offering a means to assess resource trade-offs and constraints through a nexus perspective, ultimately enabling intelligent optimization of energy efficiency.

However, it is important to acknowledge the limitations of this study and address them in future research endeavours. One limitation lies in the data used for the model. More specific surveys focusing on the consumption patterns of each end use, as well as the interactions between food consumption and the use of food-related electric appliances, would enhance the accuracy and reliability of the model. To further advance our understanding of the household FEW nexus, exploring additional factors that influence resource interactions and quantifying their impact on FEW consumption would provide a more comprehensive perspective.

## Data availability

Data will be made available on request.

## CRediT authorship contribution statement

**Hanyu Liu:** Writing – review & editing, Writing – original draft, Visualization, Validation, Software, Resources, Methodology, Investigation, Formal analysis, Data curation, Conceptualization. **Wanglin Yan:** Writing – review & editing, Writing – original draft, Visualization, Resources, Project administration, Investigation, Funding acquisition, Conceptualization. **Hikaru Kobayashi:** Supervision, Resources, Project administration, Investigation, Conceptualization.

## Declaration of competing interest

The authors declare that they have no known competing financial interests or personal relationships that could have appeared to influence the work reported in this paper.
