# Supplementary material for: Modelling the system dynamics of household food, water, and energy nexus effects
Source: Heliyon. 2024 Feb 12;10(4):e25886. doi: 10.1016/j.heliyon.2024.e25886 (PMC10884418; doi:10.1016/j.heliyon.2024.e25886)
Supplement: Multimedia component 1 [file mmc1.docx]

Supplementary information

**S1 System dynamics model with FEW nexus for houses with resource self-production systems**

This system dynamics (SD) model has four main components, including energy, water, food, and nexus interactions. In the energy subsystem, the energy consumption of each end-use appliance constitutes the household energy demand. The appliances are divided into lighting, water heating, wet appliances, space heating and cooling, and food processing & storage according to their purposes. The main factors influencing energy demand are ownership, efficiency, and duration of usage of each appliance. On the other hand, energy self-production systems include photovoltaic system, solar thermal system. The factors that affect the performance of energy self-production systems are mainly weather factors, including radiation and temperature. In this study, the relationship between the performance of energy self-production systems and weather factors was first evaluated by the regression analysis based on historical data, and then applied to the SD model.


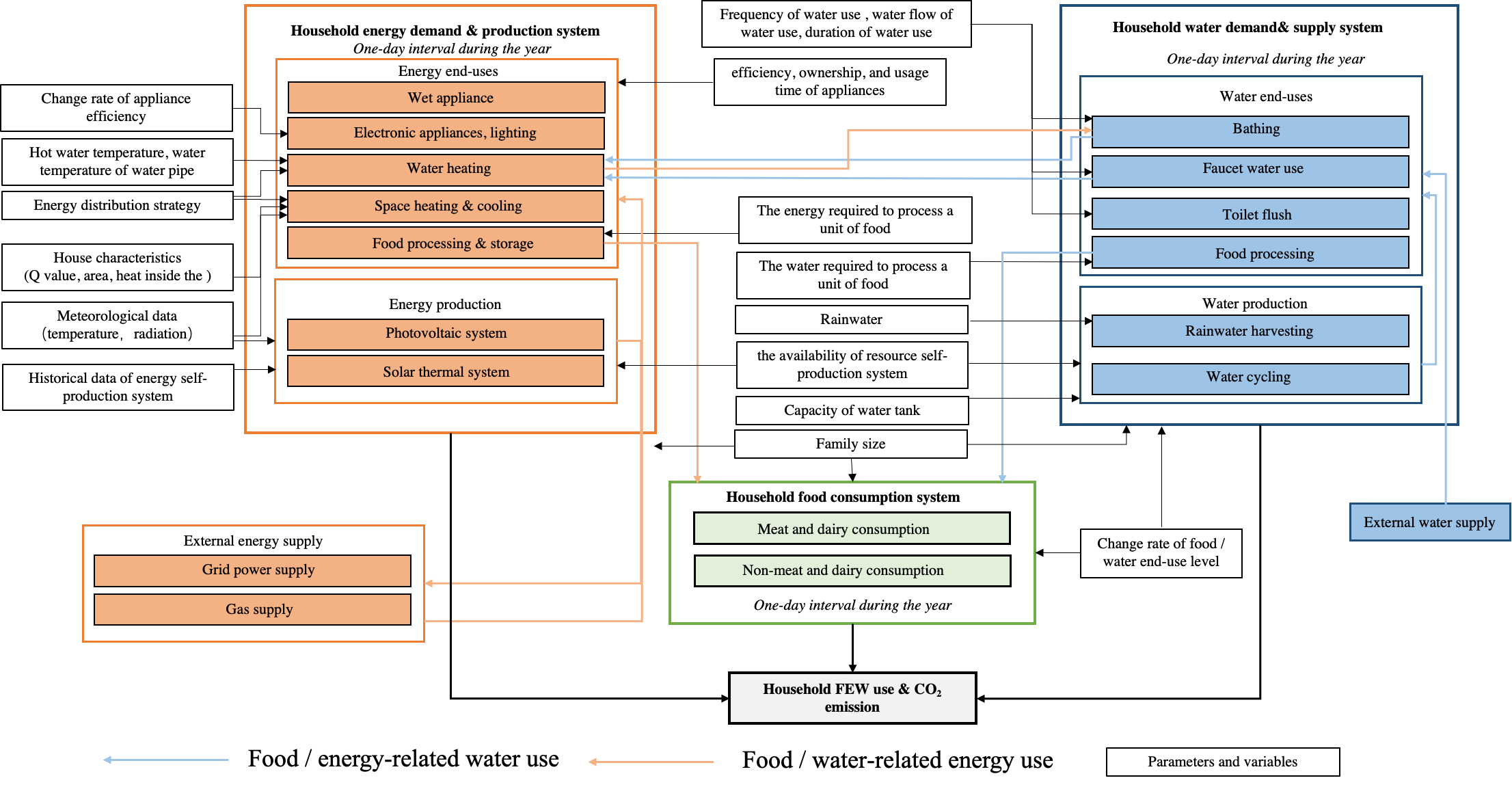


Fig S1 Framework of household FEW nexus model

In the water subsystem, bathing, faucet water use, toilet flushing, and food processing make up the household water demand. Factors affecting each water use include frequency of water use, duration and flow rate of each water use. The water subsystem also includes water recycling and rainwater systems, and factors that influence these include rainfall and water tank capacity.

In the food subsystem, the energy and water use caused by food processing and food consumption were estimated based on different food types. The influencing factors are mainly the energy and water use per unit of food processing for each food category. In the nexus interactions subsystem, the interactions in the household FEW nexus and the CO2 emissions due to FEW consumption are figured out.


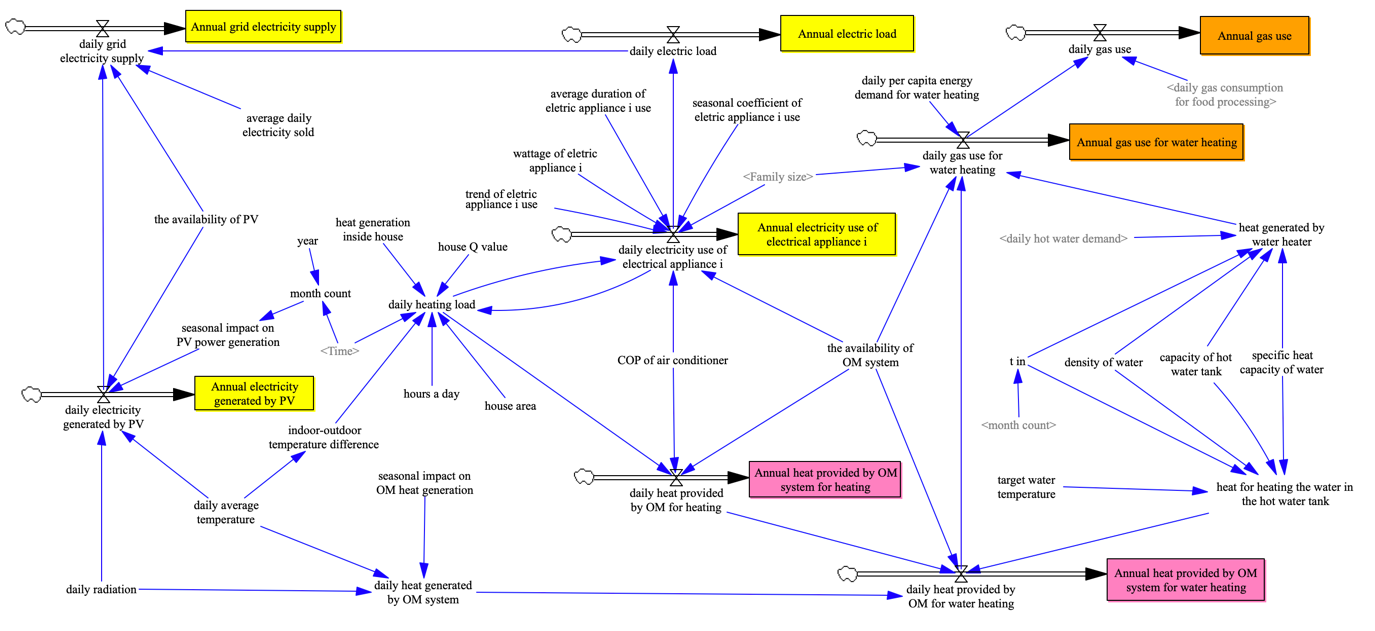


(a) The stock and flow diagram of household energy system


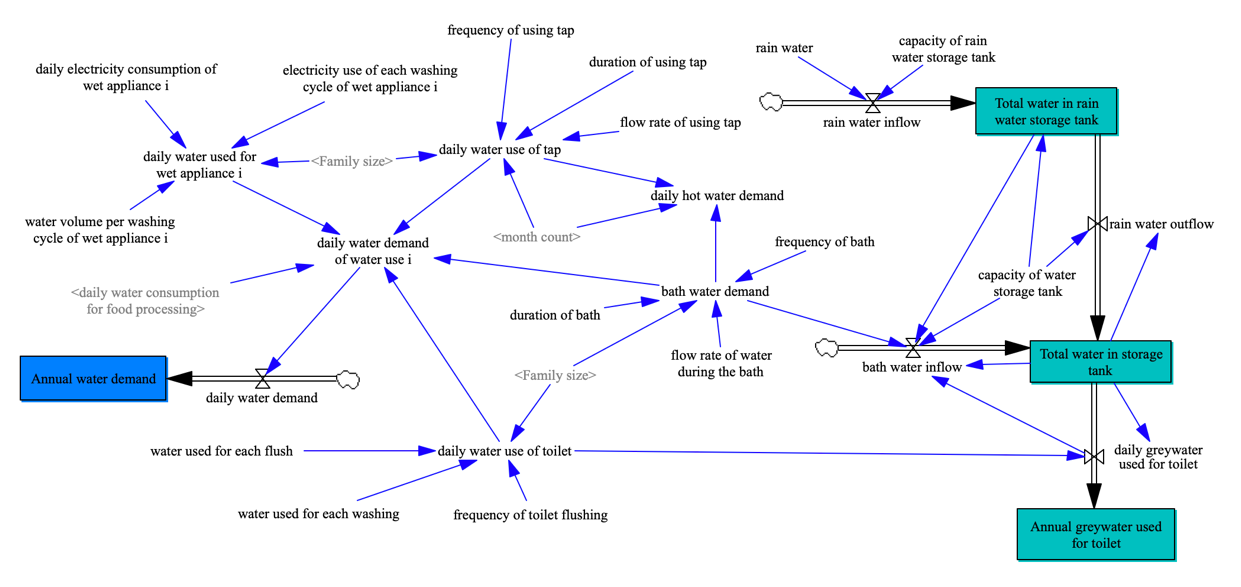


(b) The stock and flow diagram of household water system


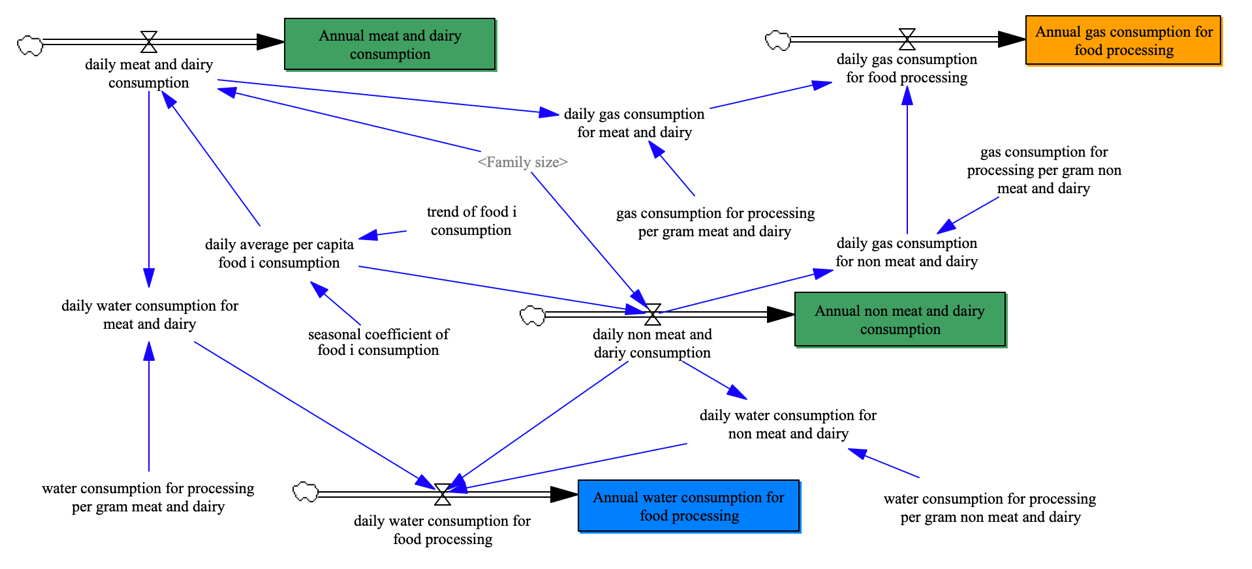


(c) the stock and flow diagram of household food system


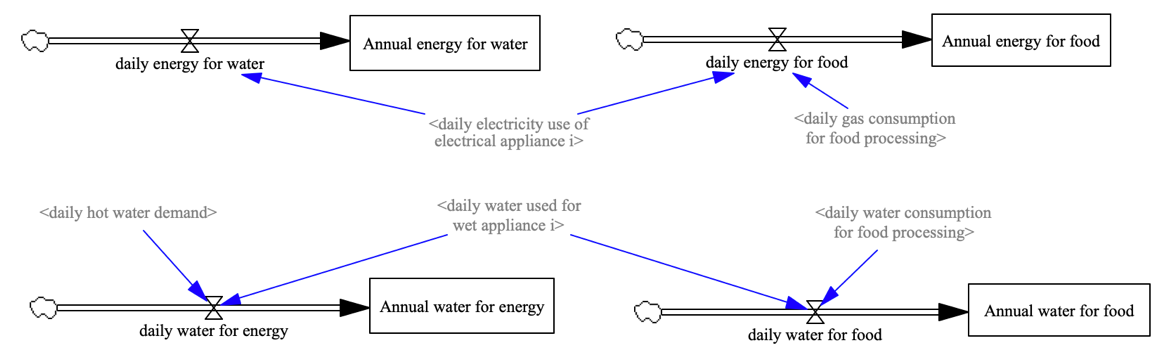


(d) The stock and flow diagram of nexus interactions between FEW use

Figure S2 The stock and flow diagram of household FEW nexus dynamics model

**S2 Input parameters of household FEW nexus model**

Table S1 Input parameters of water subsystem

Table S2 Input parameters of energy subsystem

Table S2 input parameters of food subsystem

**S3 Equations for household FEW nexus model**
